# Towards predicting implant-induced fibrosis: A standardized network model of macrophage-fibroblast interactions

**DOI:** 10.1016/j.csbj.2025.07.022

**Published:** 2025-07-13

**Authors:** Matilde Marradi, Martijn van Griensven, Nick R.M. Beijer, Jan de Boer, Aurélie Carlier

**Affiliations:** a Department of Cell Biology–Inspired Tissue Engineering, MERLN Institute for Technology-Inspired Regenerative Medicine, Maastricht University, P.O. Box 616, Maastricht, MD 6200, the Netherlands; b National Institute of Public Health and Environment, Centre for Health Protection, Antonie van Leeuwenhoeklaan 9, Bilthoven, MA 3721, the Netherlands; c Department of Biomedical Engineering and Institute for Complex Molecular Systems, Eindhoven University of Technology, Eindhoven, the Netherlands

**Keywords:** Foreign body response, Mechanotransduction, Immune response, Fibrotic tissue, Standardized ordinary differential equations

## Abstract

The foreign body response (FBR) is a complex and multifaceted process that remains incompletely understood, often leading to complications in medical device integration. In this study, we constructed a literature-based network of the FBR and developed it into a semi-quantitative predictive model to better understand its dynamics. The *in silico* FBR model incorporates key material-related factors, including immunogenic properties and mechanical mismatch, which influence immune cell activation and extracellular matrix (ECM) deposition. Predictions align with existing knowledge, showing that material stiffness and tissue progressive stiffening due to increased ECM deposition can exacerbate the FBR and that feedback interactions can protect the system from pathological outcome by gradually reducing the initial inflammatory input. The model also successfully replicated six out of eight experimental cases of anti-fibrotic interventions, demonstrating its potential as a predictive tool. Assessing implant safety in the early pre-clinical stages of device development is critical for ensuring long-term functionality and reducing adverse reactions. By systematically analyzing and integrating all interacting aspects of the FBR, *in silico* modeling can provide valuable insights and complement *in vitro* and *in vivo* studies for improved implant safety assessment.

## Graphical Abstract

**Figure:**
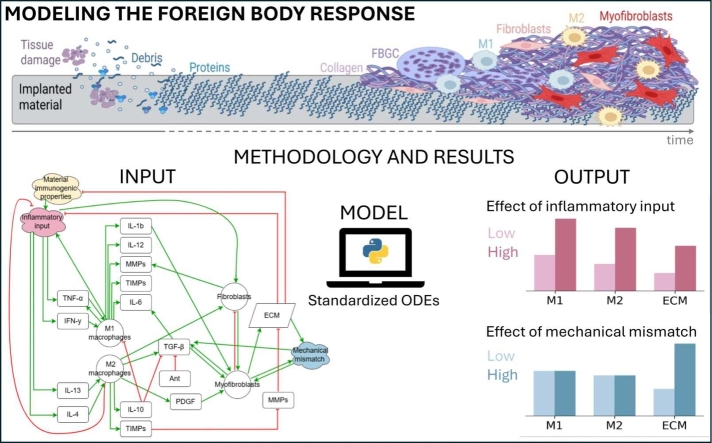

## 1 Introduction

The use of implantable biomaterials has become an essential part of modern medicine, with increasing utilization each year [1]. Those biomaterials include for example sutures, structural meshes, soft tissue fillers, orthopedic prosthetics, vascular stents, valvular prostheses, biosensors, contraceptive devices, and long-term drug-eluting devices. The success of these devices depends on the extend/severity of the foreign body response (FBR) [2]. Specifically, the immune system responds to the prolonged presence of a foreign material, and induces the formation of a capsule-like dense fibrous tissue that isolates the device or material. Depending on the site of implantation, the type of implant and the patient, the FBR and subsequent fibrotic encapsulation can manifest across a spectrum of severity, potentially leading to negative health effects and implant failure. For example, the failure rate of breast implants alone due to the FBR is 30 %, and if the failure rate of all other implantable devices for the same cause is conservatively estimated to be 10 %, FBR costs 10 billion US$ worldwide [3]. Mitigating the FBR to implantable medical devices is challenging due to the limited knowledge of the complex mechanisms underlying its drivers and patient-specific outcomes, as well as a poor translation of pre-clinical data (*in vitro*/*in vivo*) to humans.

*In vitro* biocompatibility studies are predominantly conducted in 2D cell culture systems, which can only partially replicate the complex three-dimensional and interconnected physiological environment [4]. For example, it has been shown that macrophages in these 2D models exhibit phenotypes and responses that differ significantly from those observed *in vivo* [5]. Additionally, short-term biocompatibility assessments (lasting less than a month) may yield biased results. The prolonged (weeks to years) evolution of the FBR makes it impractical to experimentally assess implant performance *in vitro* under diverse conditions over extended periods. Long-term *in vivo* studies are limited in replicates and provide only limited insight into intermediate stages, as detailed temporal data are often inaccessible. Moreover, choosing an appropriate animal model to replicate the FBR in humans is crucial, as different species and strains can elicit markedly distinct FBR outcomes, potentially limiting human translation [6].

Considering that the FBR is shaped by a complex interplay between biomaterial properties, the immune system, fibroblasts, and extracellular matrix dynamics, where unresolved pathological signals tip the balance toward chronic fibrosis, addressing FBR-related issues associated with implantable medical devices requires a comprehensive analysis of these interacting factors using advanced and predictive methodologies, a challenge that is difficult to tackle using only *in vitro* or *in vivo* approaches.

Computational models offer a systematic methodology to decouple the effects of multiplexed stimuli, non-linear interactions and feedback loops between various mechanical, biochemical and biological signals [7]. This enables models to explain seemingly conflicting *in vitro* or *in vivo* results, and produce testable hypotheses for experimental investigation of, for example, the role of biomaterials in the FBR. Once calibrated and validated with experimental data, computational models can bridge the gap between *in vitro* and *in vivo* studies by enabling long-term simulations, providing predictive insights into FBR progression, and offering a more detailed understanding of its dynamics over time. They could also serve as a valuable tool in pre-clinical testing, aiding in biomaterial performance prediction and refining safety assessments.

In the broader context of healing and fibrotic diseases, many equation-based models have been developed. Trejo *et al.* developed a model of inflammation at the early stages of the bone fracture healing process to show and test the relevance of macrophages in the fracture healing outcome. The model found that alternatively activated macrophages increase tissue production and anti-inflammatory cytokines, which improves the healing process in severe fractures but not in simple ones [8]. Hao *et al.* developed the first model of idiopathic pulmonary fibrosis using partial differential equations to account for cytokine diffusion in a 2D domain and used it to simulate the effect of different drugs and treatments. The model from Hao showed that pirfenidone could be effective in stopping, or even slowly decreasing idiopathic pulmonary fibrosis [9]. Other computational models focused on the response to implanted biomaterials. For example, Yang *et al.* developed a 2D model of the FBR to subcutaneous implantation of polyethylene catheters in rats and showed that a response dominated by TGF-β secreting macrophages induced earlier and higher cell proliferation [10]. Similarly, Su *et al.* modelled the FBR kinetics and found that fibroblasts activity was very significant for the risk of pathological outcome. More recently Dyck *et al.* published an illustrative model of capsular contracture in breast implants, and qualitatively showed that the entity of the feedback between collagen levels and cells recruitment and activation is key to the normal versus pathological response in a macrophage/fibroblast/collagen model [11]. All mentioned models, predominantly based on ordinary differential equations (ODEs), need extensive parameter estimation and fine-tuning to provide quantitative insights, often requiring tissue-specific experimental data. For the context of the foreign body response, the limited availability of experimental data poses challenges in estimating parameters for the numerous variables involved.

Moreover, to the best of our knowledge, the above FBR models describe the material as a debris variable that enhances early inflammation and is removed by macrophages, or as an increased macrophage recruitment parameter, without considering other effects of the material, such as mechanical, chemical [12], dimensional [13], [14], or surface properties [15], [16]. Indeed, as reviewed by Klopfleisch and Jung [2], material properties play a fundamental role in the FBR, influencing both the early inflammatory response and long-term fibrotic outcomes. For example, surface characteristics such as wettability, topography, chemical composition, and charge affect the adsorption of plasma proteins, which in turn modulates immune cell recruitment and activation [17]. This adsorption can be modulated by altering surface chemistry, for example through protein-repellent coatings or engineered functional groups such as triazine derivatives [12]. Another aspect that modulates the immune response in a context-dependent manner is the mechanical mismatch between the stiffness of the implant and that of the surrounding tissue. Stiff implants in soft tissues generate stress at the interface during physiological movement, and mechanical stimuli have been recently demonstrated to induce the activation of fibroblasts into myofibroblasts [18], [19].

Through the present computational modeling work, we aim to enhance the understanding of the driving mechanisms and complex interactions of the FBR and elucidate the role of material properties and mechanical cues in shaping its outcome. Hereto, we propose a semi-qualitative model of the FBR that avoids heavy reliance on parameter calibration. The model incorporates key cell types, cytokines, and proteins alongside the most important feedback loops, forming a comprehensive, literature-based network. We created three independent input variables to investigate the effects of material properties on the pathological outcome, focusing on material stiffness and immunogenic properties. Using standardized differential equations and homogeneous parameter values, our model emphasizes fundamental insights from structural interactions, independent of the parameter values. Specifically, it shows that material stiffness and tissue progressive stiffening due to increased ECM deposition can exacerbate the FBR and that feedback interactions can protect the system from pathological outcome by gradually reducing the initial inflammatory input. Ultimately, *in silico* models like ours provide new avenues to bridge the gap between *in vitro* and *in vivo* studies, offering a robust framework for designing biomaterials with improved biocompatibility and long-term performance in the early stage of device development to pave the way for clinical translation.

## 2 Materials and methods

The aim of our work was to develop a dynamic network model of the core processes of the FBR, providing insights into the impact of inflammatory signaling due to tissue injury immediately after implantation, along with the immunogenic and mechanical properties of the implanted biomaterials, on the long-term interactions that may arise at the implant site.

In the following sections, we detail the steps involved in creating this model, including the derivation of the network structure from literature, the introduction of input variables to represent the material properties, the mathematical framework underpinning the model, and its implementation.

### 2.1 Network structure

**Fig. 1:**
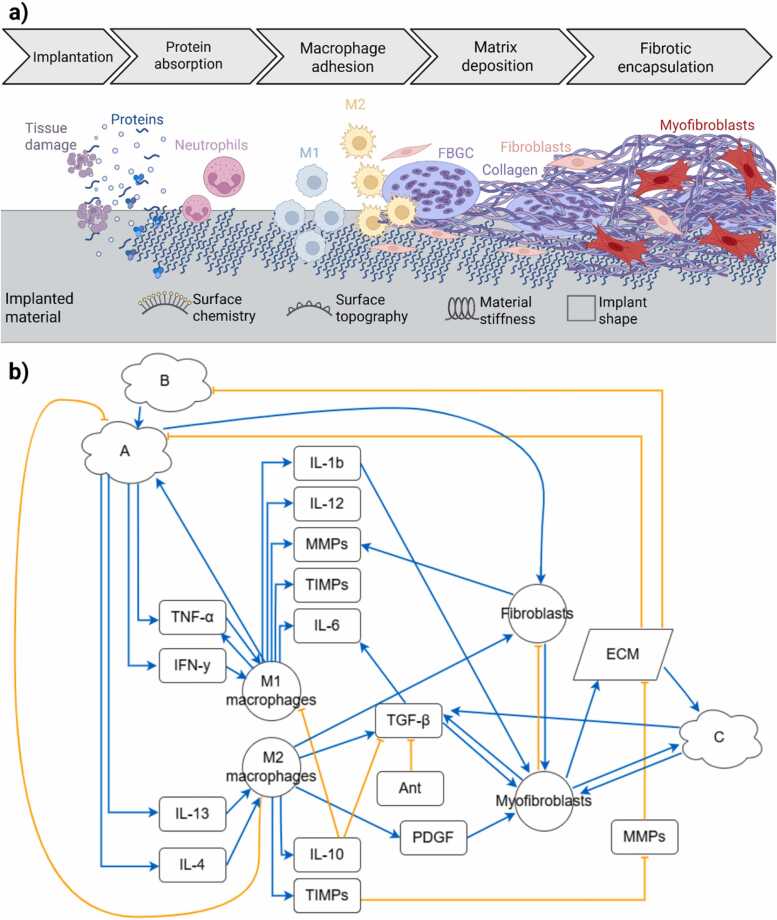
a) Schematic overview of the different phases of the foreign body response (FBR) highlighting main cell types involved and material characteristics. Abbreviations: M1 (macrophages of type 1), M2 (macrophages of type 2), FBGC (foreign body giant cells). b) Literature-based network of processes involved in the FBR. The network represents all variables included in our in silico model and their interactions as described in the literature. Sharp blue arrows indicate interactions leading to activation or an increase in the target variable, while blunt orange arrows represent inhibitory effects or decreases in the target variable. Abbreviations: TNF-α (tumor necrosis factor-α), IFN-γ (interferon-γ), IL- (interleukin-), M1 (macrophages of type 1), M2 (macrophages of type 2), MMPs (matrix metalloproteinases), TIMPs (tissue inhibitors of metalloproteinases), TGF-β (transforming growth factor-β), PDGF (platelet derived growth factor), ECM (extracellular matrix). Ant is used to indicate other antagonists of TGF-β that were not explicitly included in the model. A indicates the input variable activating immune cells, B the input of immunogenic properties of the material and C the input variable for the mechanical mismatch in the peri-implant area (see Table 1 for a more detailed description).

A biological network was built, summarizing the prevailing view of previous experimental studies found in literature, with a specific focus on the paracrine signaling between macrophages and fibroblasts as one of the key aspects in the FBR (see Fig. 1). In particular, a literature review on the FBR in soft tissues was conducted and papers were divided into two groups: *in vitro* data to be used for defining the structure of the network and *in vivo* data to validate the *in silico* perturbation studies. The network structure was first derived from reviews on FBR. In a second stage, additional literature support, i.e. at least two independent studies, was sought for each interaction in the network to ensure consensus (see Supplementary Material Table S1 for an overview of the evidence for each network interaction). When available, data specifically on FBR was used; if no data on the FBR were present, data on the broader topic of fibrotic diseases in soft tissues was used.

The FBR begins immediately upon implantation of the biomaterial with tissue damage from opening the tissue influencing damage-associated molecular patterns (DAMPs) [20]. In addition, plasma proteins rapidly adsorb onto the biomaterial surface, forming a provisional matrix that acts as the initial interface for immune cells (see Fig. 1). This interaction, together with signals originating from the damaged tissue, triggers acute inflammation, where immune cells, such as neutrophils, arrive at the site, releasing inflammatory mediators such as interleukin 4 and 13 (IL-4, IL-13), tumor necrosis factor-α (TNF-α), and interferon-γ (IFN-γ) [21]. These inflammatory signals drive macrophage recruitment and polarization, marking the transition to chronic inflammation. We distinguish two types of macrophages, based on their polarization state: classically activated macrophages (M1) and alternatively activated macrophages (M2). Although it is generally accepted that M1 and M2 represent two polar ends of a continuum exhibiting pro-inflammatory and tissue repair activities [22], we considered this two-state simplification appropriate to avoid adding an additional degree of complexity and uncertainty to the model. Macrophages of type M1 release pro-inflammatory cytokines like IL-1, IL-6, and TNF-α to clean the wound of bacteria, foreign debris and dead cells, while M2 macrophages, stimulated by IL-4 and IL-13, secrete transforming growth factor-β (in this work, all isoforms belonging to the superfamily are referred to as TGF-β), platelet derived growth factor (PDGF), vascular endothelial growth factor (VEGF), and enzymes belonging to the family of matrix metalloproteinases (MMPs) that promote anti-inflammatory effects and tissue growth [21]. A too prolonged or unbalanced activity of macrophages is believed to exacerbate fibrosis [23], [24]. One of the hallmarks of fibrosis is the presence of foreign body giant cells (FBGC), multinucleated cells originating from the fusion of macrophages, that secrete degradative species, which can induce damage to the material or facilitate its degradation if the biomaterial is degradable [25].

The network also includes fibroblasts and myofibroblasts (see Fig. 1). Fibroblasts migrate to the site and are activated by cytokines such as members of the TGF-β superfamily and PDGF, as well as by mechanical signals from the implant environment into myofibroblasts [26], [27]. Myofibroblasts are characterized by increased secretion of extracellular matrix components (ECM) and by the presence of stress fibers incorporating α-smooth muscle actin (αSMA). Due to αSMA fibers they exert contractile forces that induce mechanical strains in the tissue [28]. In our model, we capture the transition from fibroblasts to myofibroblasts. After being recruited from the initial inflammatory input and by macrophage cytokine secretion, fibroblasts can transition to the myofibroblast type, therefore the value for fibroblasts decreases when myofibroblasts increase. Sustained myofibroblast activation over time can lead to excessive ECM deposition and the formation of a thick fibrotic capsule around the implant [29].

The FBR network also models key individual cytokines (TNF-α, IL-4, IL-10, IL-13, IL-1b, IL-6, IFN-γ), families of growth factors (referred to as TGF-β and PDGF) and the family of MMPs as only one variable, as well as their inhibitors (collectively referred to as TIMPs).

Notably, the network interactions represent a combination of multiple biological processes lumped together. As such, they act as proxies for the broader dynamics of the FBR, enabling investigation of system level behavior. Our aim was to build a standardized ODE model (detailed in Section 2.2); consequently, all interactions were simplified to one of two possible actions: activation (sharp arrow) and inhibition (blunt arrow), neglecting how these actions could switch over time. In the case of TGF-β, we included inhibition by IL-10 together with a lumped inhibitor (Ant in Fig. 1) accounting for all antagonists of TGF-β production and activation that could not be explicitly incorporated into our study. This approach reproduces partial inhibition by IL-10 without adding excessive complexity to the model. Nakagome *et al.* showed that deletion of the IL-10 gene in explanted alveolar macrophages increased TGF-β production to one third of wild type [30]. To match this finding the Ant node was set to a constant value of 0.3.

In the model TGF-β has two activators (M2, mF) and two inhibitors (IL-10, Ant). The increase of either M2 or mF increases TGF-β and an increase of IL-10 or Ant will decrease it. Note that in the dynamic model (see Section 2.2), the various interactions get integrated so that multiple inhibitors may overrule the activators. As the strength of the activation and inhibition dynamically changes, the standardized ODE framework does capture, albeit limitedly, context-dependent effects where the same interactions can lead to multiple outcomes. The FBR network focuses on two cell populations, macrophages and (myo)fibroblasts, and the most relevant cytokines and growth factors. While these cells are currently recognized as the central drivers of the FBR, it is important to acknowledge that many other cell types, particularly other immune cells, and additional cytokines contribute to the complexity of the process.

#### 2.1.1 Input variables

The FBR starts with the implantation procedure that damages surrounding tissues and activates the immune response and we represented these initiating events using three independent input variables. Since our interest was to investigate the material influence on the process, we divided the three independent inputs into two categories: wound-related (A), which would occur even without the material, and material-related (B and C). Notably, these variables do not correspond to specific material properties or quantifiable surgical variables (e.g. incision size) in a one-to-one manner. Rather, they lump together many aspects of the complex peri-implant area. See Table 1 for a more detailed description of the assumptions and processes lumped together in these input variables.

**Table 1:** Summary of the interactions of input variables A, B, C with other variables in the network.

Input variable	Meaning	Translation in the network
A	A is the activating event for immune cells, it induces secretion of cytokines that polarize macrophages, therefore it lumps all the inflammatory events of the very early phase, from insult to platelet activation, damage associated molecular patterns, neutrophils recruitment [21]. A also induces fibroblasts recruitment, which happens simultaneously with early inflammation, due to the disruption of tissue continuity [31].	Activating arrow from A to all cytokines Activating arrow from A to fibroblasts
B	B enhances A, lumping the immunogenic properties of a generic implant material, that induces activation of the immune system, therefore enhancing the inflammatory cascade events from A [32]. B does not decrease over time, because it represents a non-degradable material meant for long-term use.	Activating arrow from B to A
C	C represents the local mechanical stimuli, which are induced by the stiffness mismatch with respect to native ECM at the implantation site (soft tissues) and, if present, by αSMA stress fibers contraction and excessive collagen deposition [33]. C influences myofibroblasts activation directly (intrinsic mechanism) and via latent-TGFβ mechanical activation (extrinsic mechanism) [34], [35].	Activating arrow from C to myofibroblasts Activating arrow from C to TGF-β

To capture the dynamic changes that influence the input variables over time, A, B and C were made dependent on other variables in the FBR network. Therefore, the initial prescribed value gets updated over the simulated time. For instance, when anti-inflammatory macrophages are present at the wound site and engulf cellular debris, the inflammatory stimulus A, which recruits immune cells, is attenuated. To obtain this effect, auxiliary variables were added to the dynamic model, as explained in paragraph 2.2. The biological rationale underlying each feedback loop acting on the input variables is provided in Table 2.

**Table 2:** Summary of the feedback effects affecting the input variables A, B, C and the assumptions on which the feedback actions were based.

Input variable	Feedback arrow in the network	Meaning
A	Activating arrow from M1 to A	Pro-inflammatory action of macrophages of type M1.
	Inhibiting arrow from M2 to A	Anti-inflammatory action of macrophages of type M2 reduces the level of activation of the inflammatory input variable A.
	Inhibiting arrow from ECM to A	ECM deposition at wound site concurs to restore tissue continuity, thus reducing the initial inflammatory input A.
B	Inhibiting arrow from ECM to B	ECM deposited at wound site surrounds the implant, hiding the non-self material from the immune system. The lumped effect is that ECM deposition reduces the immunogenic input B.
C	Activating arrow from ECM to C	Sustained ECM deposition (see Table 3 for threshold parameter) results in formation of fibrotic tissue, increasing the local stiffness of the tissue.
	Activating arrow from mF to C	High presence of αSMA fibers (see Table 3) allows for increased tissue contraction, increasing the local stretch and stiffening the tissue.

### 2.2 From the FBR network to a dynamic model

The network structure was converted into a dynamic computational model using the standardized ordinary differential equations method introduced by Mendoza and Xenarios [30]. This method semi-quantitatively calculates the steady state activation levels of all variables in the network. The advantage of standardized equations is that each equation is defined by the same formalism (function). In addition, all parameters are assigned the same value. This results in a simplified model, but also less affected by parameter uncertainties, which is beneficial given the limited quantitative experimental data available on the FBR. The disadvantage is that the model does not capture the temporal behavior, but it allows identification of the system’s stable steady states.

The standardized equations are structured as follows: for each node *i* in the network, its normalized activation level is denoted by $x_{i}$. The differential equation for $x_{i}$ over time consists of two terms: an activation term and a decay term (see Eq. 1). For simplicity, the decay term is directly proportional to the activation level of the node (i.e., $y_{i}$=1). The activation term is represented by a sigmoid function of omega (ω), which denotes the total input to the node. The sigmoid passes through the points (0,0), (0.5, 0.5) and (1,1), regardless of the value of its gain, h. Consequently, all variables are defined within the [0,1] interval. In sigmoid functions, higher gain values induce a steeper increase. Omega is a combination of multiple activation and inhibitory interactions on node *i* (see Eq. 2). All alpha (*α*) and beta (*β*) parameters were assigned a default value of 0.12 and the gain parameter (h) a value of 1. In the supplementary material we report the results for different values of parameter h. When data becomes available, the framework allows for modification of the parameter values to fine-tune the behavior of the equations. Note that, given the quasi-linear behavior of the system in its baseline version with h= 1, varying the initial conditions of the intermediate variables (randomly) does not influence the steady state activation levels (results not shown).

(1) $$\frac{dx_{i}}{\mathrm{dt}}=\frac{-e^{0.5h}+e^{-h\left(\omega_{i}-0.5\right)}}{\left(1-e^{0.5h}\right)\left(1+e^{-h\left(\omega_{i}-0.5\right)}\right)}-\gamma_{i}x_{i}$$

(2) $$\omega_{i}=[\begin{array}{c} \left(\frac{1+\sum \alpha_{n}}{\sum \alpha_{n}}\right)\left(\frac{\sum \alpha_{n}x_{n}}{1+\sum \alpha_{n}x_{n}}\right)\left(1-\left(\frac{1+\sum \beta_{m}}{\sum \beta_{m}}\right)\left(\frac{\sum \beta_{m}x_{m}}{1+\sum \beta_{m}x_{m}}\right)\right)\;\;\&\;\\ \left(\frac{1+\sum \alpha_{n}}{\sum \alpha_{n}}\right)\left(\frac{\sum \alpha_{n}x_{n}}{1+\sum \alpha_{n}x_{n}}\right)\;\;\&\&\\ \left(1-\left(\frac{1+\sum \beta_{m}}{\sum \beta_{m}}\right)\left(\frac{\sum \beta_{m}x_{m}}{1+\sum \beta_{m}x_{m}}\right)\right)\;\;\&\&\& \end{array}$$

$$h,\alpha_{n},\beta_{m},\gamma_{i}>0$$

$$0\leq \omega_{i}\leq 1$$

$$0\leq x_{i}\leq 1$$

$$\left\{x_{n}\right\}\;is\;the\;set\;of\;activators\;of\;x_{i}$$

$$\left\{x_{m}\right\}\;is\;the\;set\;of\;inhibitors\;of\;x_{i}$$

$$\&\;is\;used\;if\;x_{i}\;has\;activators\;and\;inhibitors$$

$$\&\&\;is\;used\;if\;x_{i}\;has\;only\;activators$$

$$\&\&\&\;is\;uesd\;if\;x_{i}\;has\;only\;inhibitors$$

As mentioned in paragraph 2.1.1, the values over time of input variables A, B, and C depend also on other variables in the network (see Table 2). Implementing such dependency in the current mathematical framework required careful consideration, as according to the standardized ODE formalism, a reduction in an inhibitory input would lead to an increased activation, which is not physiological for the input variables. To address this, we introduced three auxiliary variables ($A_{\mathrm{aux}},\;B_{\mathrm{aux}},\;C_{\mathrm{aux}}\;)$ that represent the network effect on the input variables. Each auxiliary variable is computed according to the standardized ODEs, following the effects mentioned in Table 2. Consequently, the auxiliary variables, like all other variables, are defined within the interval [0,1]. Auxiliary variables are defined within the numerical implementation and depend on different points in time, therefore they are presented in the discrete form, where *t* is current iteration in the simulation (for details on the numerical implementation, see Section 2.3). At the start of each new iteration, the input variable values are updated by combining their initial value ($A_{0},\;B_{0},\;C_{0}$) with the auxiliary variable ($A_{\mathrm{aux}},\;B_{\mathrm{aux}},\;C_{\mathrm{aux}}\;)$ values computed at the previous iteration (see (3), (4), (5)). For input variables A and B, the corresponding auxiliary variables are subtracted from the initial value, as the downstream effects are intended to decrease the input. Conversely, for input variable C, the corresponding auxiliary variable is added to the initial input value. The parameters f_i_ were set to 1 (see Table 3), and if conditions were included to constrain input variables within the designated interval of [0,1]. Similar to the parameters *α* and *β*, the f_i_ values can be readily refined once more data on these effects is available.

(3) $$A(t)=\{\begin{array}{cc} A_{0}\;-\;{f_{A}*A}_{\mathrm{aux}}(t-1) & \mathrm{if}\;A_{0}>A_{\mathrm{aux}}(t-1)\\ 0 & \mathrm{elsewhere} \end{array}$$

(4) $$B(t)=\{\begin{array}{cc} B_{0}-{f_{B}*B}_{\mathrm{aux}}(t-1) & \mathrm{if}\;B_{0}>B_{\mathrm{aux}}(t-1)\\ 0 & \mathrm{elsewhere} \end{array}$$

(5) $$C(t)=\{\begin{array}{cc} C_{0}+f_{C}*C_{aux}\left(t-1\right) & if\;(C_{0}+C_{aux}\left(t-1\right))\leq 1\\ 1 & elsewhere \end{array}$$

**Table 3:** Parameter values. List of parameter values and names used in the model.

Parameter name	Parameter value
$\alpha_{n}$ – relative weight parameter value for all activators in all equations	0.12
$c_{x}$ - relative weight parameter value of the variable C	0.12
$b_{x}$ – relative weight parameter value of the variable B	0.12
$\beta_{m}$ - relative weight parameter value for all inhibitors in all equations	0.12
$\gamma_{i}$ – decay rate for all variables	1
h - gain parameter	1
$f_{i}$ – feedback parameters ((3), (4), (5)*)*	1
Initial value of all network variables	0
ECM value at steady state set as threshold dividing normal from pathological outcome	0.35
Threshold - Minimum sum of activation levels of ECM and mF to induce increase in the C variable	0.7
Other antagonists of TGF-β activation level	0.3

Table 3 provides an overview of parameter values used, while all the equations are available on Github [repository link will be made public upon publication].

The output of the model is, for each variable, a value between [0,1] that indicates its activation level. In our model the activation level represents a normalized concentration for the cell variables and ECM components. For cytokines and growth factors the activation level represents the combined effect of concentration and activity. For example, in the case of TGFβ, we lump the effect of latent-TGFβ secretion and activation through biochemical pathways and the mechanically-induced activation of the latent compound. Therefore, in the FBR model, an increased value of TGF-β can represent both increased secretion and increased activation of this growth factor. For some of the analysis presented in this work, we divided outcomes into “low” and “high” based on the steady state level of the ECM variable. We defined high (pathological) outcomes, which mimic the clinical outcome of interest (i.e., fibrotic capsule thickness around the implant), as having a steady state ECM value above 0.35. This threshold was chosen because it corresponds to the value reached for a high input A and null material-related inputs (B, C).

### 2.3 Numerical implementation

The computational model was implemented in Python 3.11, utilizing the scipy.integrate.solve_ivp function to numerically solve the system of ODEs. For the baseline version of the model, since the equations are non-stiff, we used the default explicit Runge–Kutta method (RK45, rtol= 1e-3, atol=1e-6). When performing the sensitivity analysis, some parameter perturbations produced stiff trajectories; these were handled with the adaptive LSODA integrator, which automatically switches between non-stiff (Adams) and stiff (BDF) schemes and setting the relative and absolute tolerance to 1e–9. This ensured high precision in the numerical integration while keeping the computational cost low. Additional libraries, including NumPy for matrix operations and Matplotlib for visualization, were also employed to facilitate data analysis and model visualization. The code and detailed documentation are publicly available on GitHub [https://github.com/carliercomputationallab/FBR_standardizedODEs].

### 2.4 Perturbation studies

To evaluate the robustness of the FBR model and validate its input-output relationships, the model was perturbed to mimic *in vivo* experiments found in literature. Eight *in vivo* experiments testing different antifibrotic strategies involving one or more variables present in the network were selected. Each antifibrotic strategy was replicated in the model by setting the affected variable to a fixed value throughout the simulation. For example, when a TGF-β inhibitor was tested, the TGF-β variable was set to zero, while in the case of IL-10 supplementation, the IL-10 variable was set to be equal to one over the entire simulated time. The outcomes of these perturbation studies were analyzed by comparing the steady state level of the ECM variable with the value for the baseline model (for corresponding input values). These perturbation studies were done for different initial input conditions and compared to data from literature. Five conditions (IL-4 eluting coating, TGF-β inhibitor, IFN-y gene disruption, drug coating and macrophage depletion) were based on studies on the FBR, while three studies (IL-10 supplementation, PDGF receptor blocker, MMP-8 inhibition) were more generally on fibrotic diseases. In the latter case, we only compared the conditions with material-related inputs equal to zero. A detailed summary of the perturbation studies is presented in Table 4 in Section 3.1, while additional information on the *in vivo* studies and the selection criteria is provided in Tables S2 and S3 in the Supplementary Material.

**Table 4:** Summary of the perturbation studies. The table shows the in vivo experimental conditions that were replicated with the in silico model and the results obtained in terms of decreased or increased ECM value at the steady state. Arrow down: the perturbation induced a decreased ECM steady state level with respect to baseline in the in silico model. Arrow up: the perturbation induced increased ECM steady state level with respect to baseline in the in silico model. Tick: in silico results matched in vivo findings. Cross: in silico results did not match. NC: in silico results could not be compared to in vivo findings. See paragraph 3.1 for details.

**Antifibrotic condition tested*****in vivo*****that reduced ECM deposition**		**Implant-induced fibrosis**	***In silico*****perturbation to replicate the*****in vivo***	**Effect of the perturbation on the ECM level with respect to baseline**		
			**condition**	**Input conditions**		
				A= 1, B= 0, C= 0	A= 1, B= 1, C= 0	A= 1, B= 1, C= 1
1	IL−10 supplementation [38]	No	IL−10 = 1	**↓** ✔	**↓** NC	**↓** NC
2	PDGF receptor blocker [39]	No	PDGF= 0	**↓** ✔	**↓** NC	**↓** NC
3	MMP−8 inhibition [40]	No	MMPs= 0	**↑** x	**↑** NC	**↑** NC
4	IL−4 eluting coating [36]	Yes	IL−4 = 1	**↑** x	**↑** x	**↑** x
5	TGF-β inhibitor [41]	Yes	TGF-β= 0	**↓** ✔	**↓** ✔	**↓** ✔
6	IFN-y gene disruption [42]	Yes	IFN-γ= 0	**↓** ✔	**↓** ✔	**↓** ✔
7	Inhibitory drug coating [43]	Yes	IL1, IFNy, TNFa, IL6 = 0	**↓** ✔	**↓** ✔	**↓** ✔
8	M depletion [37]	Yes	M1, M2 = 0	**↓** ✔	**↓** ✔	**↓** ✔

### 2.5 Feedback loops testing

In order to test the effect of the feedback loops detailed in Sections 2.1.1 and 2.2, we modified the baseline code by removing the effect auxiliary variables, so that the input signals would remain constant and equal to the starting value throughout the entire simulation. This version of the code is also available on the GitHub repository.

### 2.6 Sensitivity analysis

We investigated the effect of all parameters on the outcome separately (one-at-a-time sensitivity analysis). First, we tested the effect on the ECM level at the steady state of a twofold variation of all network-related parameters (see Table 3). Next, we tested the effect of a 10 % variation of the input-related parameters (fa, fb, fc, threshold, see Table 3). In this case our interest was the robustness of the effect of inputs B and C on the ECM levels, therefore the quantity of interest was the variation in ECM levels at the steady state between two conditions, one with inputs A= 1, B= 0, C= 0, and one with A= 1, B= 1, C= 1. Lastly, the gain parameter h was increased from one up to 50 to investigate the effect of a steeper growth curve.

## 3 Results

### 3.1 A predictive computational model of the FBR

Fibrotic scarring resulting from the foreign body reaction can obstruct the integration and functionality of the implanted device. Here we develop and analyze a computational model to improve the mechanistic understanding of the FBR, with a particular focus on the paracrine signaling between macrophages and (myo)fibroblasts as one of the key aspects. We propose a standardized ODE model that summarizes the consensus view of the FBR. As initiating events that trigger the FBR, we distinguish between the initial tissue damage and inflammatory activation (A), the inflammatory properties of the material (B) and the mechanical properties of the material (C).

**Fig. 2:**
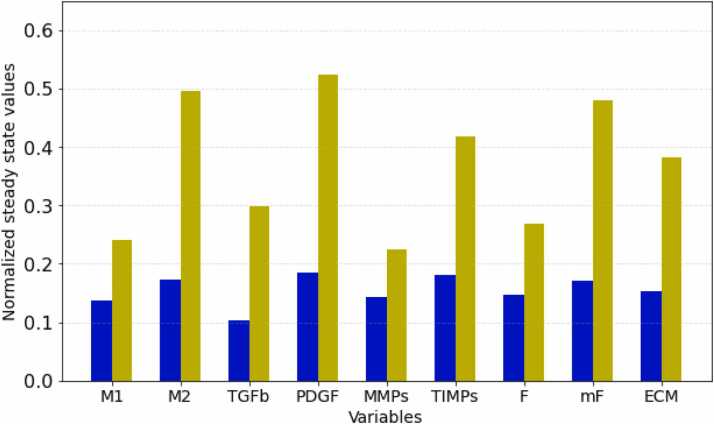
Effect of increasing the starting value of input A on the FBR network variables. Blue: A(0)= 0.3, Yellow A(0)= 0.9. In both cases B=C= 0. The normalized value at steady state between 0 and 1 represents the normalized concentration level (see Methods Section 2.2). M1 (macrophages of type 1), M2 (macrophages of type 2), TGF-β (transforming growth factor-β), PDGF (platelet derived growth factor), MMPs (matrix metalloproteinases), TIMPs (tissue inhibitors of metalloproteinases), F (fibroblasts), mF (myofibroblasts), ECM (extracellular matrix).

To first evaluate the model performance in predicting wound healing and fibrosis when excluding the implant-related input variables, we explored the effect of the initial tissue damage (A). Fig. 2 represents the relative activation values (ranging between 0 and 1) at the steady state for most relevant network variables, obtained from a baseline simulation with a low and high initial tissue damage (A), indicated in blue and yellow respectively. For the steady state values of other variables, we refer to Figure S1 in the Supplementary Material. As detailed in the methods, the activation level represents a normalized concentration. The model predictions replicate well-known *in vivo* and clinical observations, including increased activation of immune cells (M1, M2), heightened myofibroblast (mF) activation, and excessive ECM deposition with increased tissue damage (A). Note that for a maximal A input value, the corresponding steady state ECM level is just above 0.35, which we set as threshold to define fibrotic capsule formation and consequent pathological outcome. As such, the model (excluding the implant-related input variables) captures the main processes of wound healing and fibrotic ECM deposition upon excessive tissue damage.

To check the robustness and reliability of the FBR model and validate its input-output relationships, the model was perturbed in multiple ways to mimic different *in vivo* experiments. Table 4 summarizes literature, per each *in vivo* experiment replicated, the treatment tested *in vivo*, how it was mimicked in the FBR model, and the outcome in terms of variation of ECM for three different input conditions (A=1, B=0, C=0; A=1, B=1, C=0; A=1, B=1, C=1; see Table 3). As outcome we evaluated whether the difference between the steady state ECM value in the baseline and perturbed model was consistent with the experimental findings.

Note that the *in vivo* experiments on fibrotic diseases not involving an implanted material (i.e. fibrotic lung disease or skin fibrosis) could only be compared with input conditions having material-related inputs equal to zero. Specifically, the FBR model (excluding the material-related input variables) was validated against three perturbations (i.e. conditions 1–3 in Table 4, covering a wide range of processes) and correctly predicts two. The perturbation involving MMPs did not match the experimental results. Note that in the model all MMPs are lumped into one variable, that acts as an inhibitor of ECM, therefore when MMPs were knocked out, the predicted value of ECM increased.

Having set the fibrosis model in wound healing, we next explored the model performance when including the material-related input variables (B-C). The FBR model - including the material-related input variables - was validated against 5 different perturbations that covered a wide range and accurately predicted 4 (see Table 4, rows 4–8).

Since it is known that IL-4 induces M2 polarization, Hachim *et al.*, 2014 [36] tested the hypothesis whether an IL-4-releasing implant could reduce fibrotic capsule formation by promoting an early shift in the M2/M1 ratio in situ. They found that IL-4 loaded coatings of polypropylene meshes reduced the thickness of the capsule after 90 days of subcutaneous implantation in mice. They also noted that the M2/M1 ratio and IL10 levels increased. Our prediction (Table 4, condition 4) did not match the experimental results because in the *in silico* FBR model M2 macrophages only have two activators (IL-13 and IL-4). Indeed, IL-4 increases M2, as was also observed in the *in vivo* experiment [36] and results in both pro-fibrotic myofibroblast activation as well as anti-fibrotic inhibition of A. Here specifically the M2-mediated TGF-β production and myofibroblast activation is higher than the mitigating effect of increased M2 presence on resolving the inflammatory stimulus A, as the latter is regulated by more variables simultaneously.

The perturbation involving macrophage deletion matched the experimental results and also showed the relevance of the mechanical-related input C. Specifically, in the *in vivo* experiment on macrophage depletion, after implanting subcutaneous polycaprolactone (PCL) scaffolds in mice, Parlani *et al.* observed that the mice that had undergone complete macrophage depletion presented lower activation of myofibroblasts and ECM deposition, but still had some residual ECM [37]. In our simulations the knock-out of macrophages resulted in very low ECM at the steady state for all input conditions having starting value of C= 0. In these cases, the fibroblasts, being partially recruited independently of macrophages (by A directly), allowed for some ECM being produced. For increasing values of C, the ECM level further increased while always remaining below the critical threshold (i.e. <0.35; see Supplementary Figure S2), matching the partial inhibition of ECM deposition observed in the *in vivo* experiment. In the absence of macrophages, the input variable B for the immunogenic property of the material has no effect, since it is an activator of macrophages that are set to 0 for the entire simulation. In summary, the computational model captures the key processes of the FBR. Importantly, the validation accuracy was robust to a ± 50 % change in the input levels as shown in Table S4 in the Supplementary Material.

### 3.2 Interaction of inflammatory and mechanical material properties influence the FBR

**Fig. 3:**
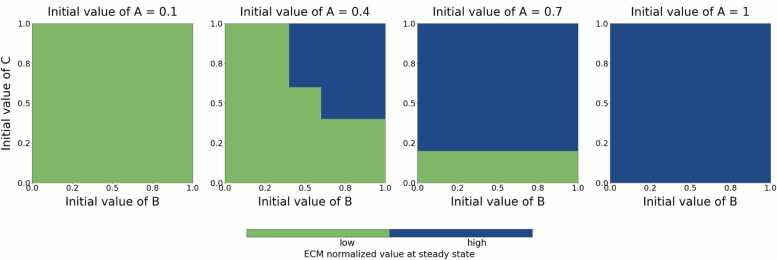
Effect of initial values of variables B and C on ECM outcomes, with varying initial values of A. The figure illustrates the influence of initial conditions for variables B and C on the normalized extracellular matrix (ECM) levels at steady state, under different initial values of variable A. Each subplot corresponds to a specific initial value of A, from the left: A = 0.1, A = 0.4, A = 0.7, and A = 0.9. The normalized ECM levels are categorized into two outcomes: low (below 0.35, shown in green) and high (above 0.35, shown in blue).

We next explored the influence of the implant-related input variables on the FBR. The results (Fig. 3) indicate that the implant-related input variables (B and C) modulate the model predictions, depending on the level of tissue damage (A). Steady state level of ECM > 0.35 (based on maximal A input value, see Fig. 2) was considered as a hallmark for pathological FBR and used here to categorize the model predictions into low and high ECM. For initial damage and immune response level A= 0.1 (Fig. 3, A=0.1), increasing the material-related inputs induces an increase in ECM but not enough to induce a high (fibrotic) state (see Supplementary Material Figure S3 for further details). For A= 0.9 (Fig. 3, A=0.9) there is high ECM outcome for all material-related input values. This result points towards a saturation effect where for extensive tissue damage fibrotic ECM production will occur in the FBR model independent of the material properties. Notably, ECM steady state levels are increasing with increasing values of B and C (see Supplementary Material Figure S3). Our model sums up the effect of three independent input variables. Consequently, the maximum input to ECM deposition is A(0)= 1, B(0)= 1, C(0)= 1 (corresponding to the top right corner of the heatmap with A(0)= 1). This condition replicates the biological scenario of very extensive tissue damage at the moment of implantation, a highly immunogenic material increasing macrophage activation, and that is also very stiff and /or shaped in such a way that induces high strains and stress to the surrounding tissue (low anatomic biocompatibility). This worst-case scenario results in a high ECM outcome, but it is not the only one.

For A< 0.8 (Fig. 3, A=0.4, A=0.7), the FBR outcome depends on the values of B and C. Implants inducing mechanical mismatch (C) and consisting of materials that cause significant inflammation (B) can induce high ECM levels.

For A= 0.4, high ECM levels are found were C> 0.5 and B> 0.5. For A= 0.7 our model also reproduces high ECM as outcome where a material has no inflammatory effect (B=0), but if the mechanical mismatch is high enough (C>0.3), the sole mechanical material properties can result in high ECM (fibrotic outcome), as was suggested to be possible by different studies on the effects of same materials with varying stiffness [44], [45]. Similarly, the model predicts that a material with maximum value for immunogenic properties (B=1) can lead to high ECM levels even if the mechanical properties of the material are equal to those of the tissue (C=0) [46]. As such, in the computational model, the FBR is shaped by the inflammatory (B) and mechanical (C) material properties alone but also through their interactions. The model predictions also indicate that the impact of the mechanical properties (C) is stronger (see Supplementary Material Figure S3), even though the parameter values have the same value for all interactions. We further evaluated the effect of parameter values of the inflammatory and mechanical material-inputs in Section 3.5. In summary, the FBR model predictions align with experimental and clinical observations that the FBR is not only determined by the extent of tissue damage but also by the mechanical and immune-related properties of the material [47].

### 3.3 Feedback loops on input variables protect from fibrotic outcome

**Fig. 4:**
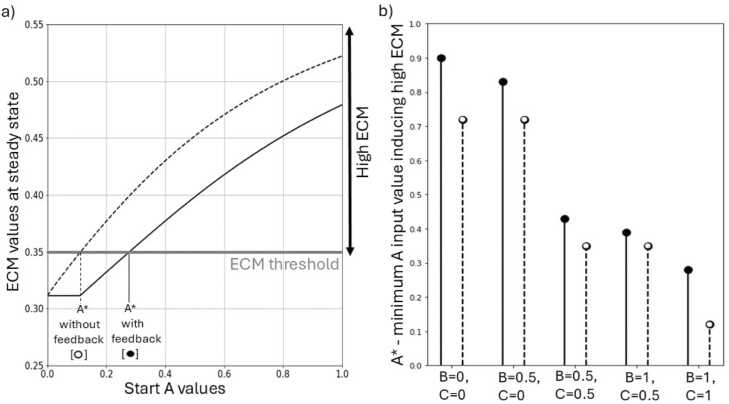
The effect of feedback loops involving input variables on the high ECM outcome in the in silico FBR model. a) steady state ECM values as a function of the initial value of the input variable A, highlighting the value of A (A*) for which the threshold of 0.35 is reached. Solid line = B= 1, C= 1, with feedback; Dotted line: B= 1, C= 1, without feedback. b) A* for different combinations of B and C input values. Solid line, solid marker: baseline model with feedback loops to input variables. Dotted line, hollow marker: model without feedback loops to input variables.

In the FBR variables influence each other in complex and intricated ways that are not entirely known yet. Here, we used the FBR model to investigate how various feedback loops affect the severity of the response. We implemented a version of the model that did not include feedback loops on the input variables A, B, C and performed simulations for multiple input initial values. In Fig. 4 we reported the minimum value of A (A*) that induces ECM above the set critical threshold between low and high ECM levels for different combinations of B and C. In all cases, removing the feedback loops results in higher ECM levels at the steady state (see Figure S4 in the Supplementary Material). Consequently, as is shown in Fig. 4, without feedback loops the system goes to high ECM levels for lower input values of A (lower A*). This behavior shows how the presence of feedback loops on the input variables protects from fibrotic outcomes in the *in silico* FBR model. Note that this effect is dependent on the variable values.

Due to how the immunogenic properties of the material (input variable B) were implemented in the model, i.e. as an activator of inflammatory input A, without feedback loops B has no effect on the model. When all interactions affecting the initial value of A are removed, A remains equal to the initial value regardless of the value of B. This is illustrated in Fig. 4, where cases that only differ for the value of B have exactly equal outcome for the simulations without feedback (same value A* in Fig. 4b, but also visible in the heatmaps reported in Figure S4 in the Supplementary Material).

### 3.4 Sensitivity analysis

We evaluated the effect of parameter values on the key findings of our model. We conducted a sensitivity analysis on the normalized parameters of the standardized framework to confirm their minimal impact. A twofold parameter variation consistently resulted in outcome changes below 10 % (see Supplementary Figure S5). Among the parameters, the most influential was cx, which is the alpha parameter of the equation for variable C (for furter detail, we refer to Eq. 2 and to the code available in the Github repository). Next, we assessed the robustness of inputs B and C to 10 % variation of non-normalized parameters (fa, fb, fc, threshold, h, see Table 3). To quantify this, we computed the percentage variation in ECM values at the steady state between a simulation with material-related inputs equal to zero and one with maximum values (i.e. input A=1, B=0, C=0 and A=1, B=1, C=1). This variation, with baseline values of all parameters was equal to 20 %, and we evaulated how it would change for varied parameter values. We found that this variation was most sensitive to the increase of the threshold parameter (See Supplementary Figure S6). The threshold parameter determines the level of myofibroblast and ECM deposition required for these two variables to affect the mechanical-related variable C via a positive feedback loop (see Methods Section 2 for more detail). Consequently, increasing the threshold reduces the variable C response. Finally, we examined the effect of varying parameter h in the model equations. While our baseline model used h= 1, other similar biological applications have employed values ranging from 1 to 25 [48], [49], [50]. In our model, h values exceeding 10 led to a more binary-like response, with reduced presence of intermediate values and oscillatory behaviour/instabilities for certain values of B and C (see Supplementary Figure S7).

Taken together, the model outcomes are very robust against network-related parameter variations. Moreover, our qualitative finding that increasing B and C increases the ECM values at the steady state is robust to variations in all input-related parameters.

## 4 Discussion

The FBR is a complex and not yet fully understood process that can lead to complications in the treatment of patients using medical devices in various applications. The FBR is extensively studied from both material and biological perspectives, with the aim of developing effective treatments and reducing the incidence of failures caused by this response. In this work, we manually reconstructed a literature-based network of the foreign body response. This network was used to develop a semi-quantitative predictive model of the FBR. The model's predictions align with the consensus that higher initial inflammation, with other factors constant, increases fibrotic encapsulation. Additionally, the model incorporates material-related inputs to capture the immunogenic properties of a non-degradable material (B) and the mechanical mismatch between the implant and native tissue, inducing stresses and strains in the peri-implant region (C). These inputs make the model specific to the FBR and distinct from models of fibrotic diseases or general tissue repair. When incorporating material-related variables, the predictions consistently show increased ECM levels across all initial values of the inflammatory response (A). The finding was robust to increases of normalized parameter values of up to two-fold.

Literature indicates that immediately following implantation, nonspecific host proteins are rapidly adsorbed onto the material surface, triggering complement activation and leading to immune cell infiltration and activation [51]. The phenomenon is captured by the model, where increasing input B leads to higher immune activation and ECM deposition. Our model predictions highlight that modifying material surface characteristics and charge, which influence protein adsorption, can reduce the FBR and evade immune recognition. Notably, for low levels of initial damage and immune response, increasing the material-related inputs induces an increase in ECM but not enough to induce a fibrotic state. This behavior is consistent with the notion that both in wound healing and the FBR, some degree of tissue deposition is necessary to restore tissue integrity and integrate the implanted material. The pathological outcome only occurs when this process is exaggerated over time and exceeds a certain threshold. This is less likely to happen when the initial injury is minor [52].

The model was perturbed to simulate eight different anti-fibrotic approaches tested *in vivo*, and the model predictions agreed with the experimental outcomes in six out of those eight cases. The number of perturbations evaluated was constrained by the availability of *in vivo* studies (see Table S2 for the selection criteria) and therefore can only be considered a partial validation of the model. Moreover, we would like to emphasize that the *in vivo* experiments are subjected to high variability due to the use of different species and cell lines, and due to the analysis of different and sparse time points and conditions that are not easily repeatable and reproducible (see Table S3 in the Supplementary Material). Nevertheless, the agreement between model predictions and experimental results demonstrates the reliability of the model.

Hachim *et al.*, 2014 found that IL-4 loaded coatings of polypropylene meshes reduced the thickness of the capsule after 90 days from subcutaneous implant in mice [36]. They also noted that the M2/M1 ratio and IL-10 levels increased. In our model the simulated addition of IL-4 induced higher M2 activation, leading to increased PDGF and IL-10, while downstream we found higher mF and ECM (interpreted as higher thickness). Therefore, our prediction aligns with experimental findings showing an increased M2/M1 ratio and elevated IL-10 levels, as observed also in another *in vivo* study [53]. However, it does not match the observed increase in TGF-β and reduction in fibrotic thickness. This discrepancy may stem from the model's assumption of constant and uniform parameter values across all processes, which does not account for the relative weight of certain actors or their potential variation over time. Specifically, studies suggest that the effect of IL-4 in promoting M2 macrophages may be transient, initially increasing the M2/M1 ratio towards a less pro-inflammatory state but failing to sustain M2 influx in the chronic phase [36], [44]. Future work should focus on quantifying these time-dependent cytokine dynamics and investigate their effects on fibrotic outcomes in a more advanced differential equation framework.

Garci’a-Prieto *et al.* observed that MMP-8 knocked-out mice showed reduced fibrotic tissue formation in bleomycin-induced lung fibrosis, an outcome that our model did not match (see Table 4). Notably, our model only considers generic MMPs as a single entity that inhibits ECM, while the *in vivo* experiment by Garci’a-Prieto *et al.* focused on the role of MMP-8 [40]. While the overall effect of MMPs is typically considered to be reducing fibrotic tissue formation, individual MMPs, such as MMP-8, can play opposing roles in the process [40]. This suggests that future work could model in more detail the specific effects of different MMPs and their influence on cytokine activation [54]. To do so, experimental data assessing different MMPs *in vitro* and *in vivo* in the context of the FBR is needed.

Parlani *et al.* observed that macrophage depletion did not completely inhibit fibrosis after subcutaneous implantation of PCL scaffolds in mice. This implicates the existence of macrophage-independent mediators. Among possible explanations, Parlani *et al.* suggested that high strain/mechanical stress induced by material implantation and implant stiffness could further modulate myofibroblast activation without affecting the number of macrophages [37]. Our model supports this hypothesis by predicting incomplete inhibition of ECM production due to fibroblasts being partially recruited independently of macrophages and due to myofibroblast activation induced by mechanical factors. Our results show that, in the absence of macrophages, the mechanical-mismatch initial input has a great impact on the ECM levels at the steady state.

Material stiffness has been shown to affect the encapsulation process, with stiffer materials typically eliciting a stronger fibrotic response than soft ones [45]. For various fibrotic diseases, increased ECM stiffness is also known to exacerbate the process [35]. Our model confirms these observations, as increasing variable C leads to elevated ECM levels. Note that, while this prediction constitutes a good proof of concept, the concept of mechanical mismatch (C) currently lumps together many aspects present in peri-implant soft tissues (e.g. material properties such as fiber size, pore size, mechanical strength and elasticity, the effect of fibroblast contraction and of stiff collagen deposition) into a qualitative, non-dimensional variable. Currently, the effect of mechanical-mismatch (C) in our model influences fibroblast to myofibroblast transition and increases active TGF-β levels (through mechanical activation of latent TGF-β [55]). Emerging research also highlights the role of substrate stiffness in macrophage activity and polarization [44], suggesting a future direction for enhancing the model by integrating this aspect. Moreover, recent work by Ward et al. found that intermittent mechanical loading can modulate the fibrotic behavior of fibroblasts, suggesting another immunomodulatory aspect of mechanical cues [56]. Future research could further develop and apply the FBR model to predict optimal mechanical stimulation regimes that reduce FBR. By understanding and simulating the role of mechanical stimulation, the in silico FBR model could aid in identifying potential compensatory mechanisms to improve the design of multimodal personalized treatments that combine mechanical stimulation with pharmacotherapy.

Our model predictions also indicate that the presence of feedback loops results in an increased capacity to withstand disruptive inputs without transitioning into a pathological state. This enhanced resilience is consistent with other observations on inflammatory processes, where negative feedback increases the robustness of the system to certain perturbations [57] and positive feedback (like the one for C) can induce bi-stability and exacerbate the process [11], [58]. Experimentally quantifying the relative strengths of different feedback loops could also help prioritize therapeutic targets. Future experimental research should focus on testing precise feedback mechanisms through targeted inhibition or stimulation of the involved pathways. One approach is to target the integrin-mediated latent TGF-β activation via inhibition of αv integrins [45]. Quantitative evaluation of the effect of this inhibition on the levels of active TGF-β can help refine the relative parameter in the equations. Another approach is to expose M0 macrophages to either M1-conditioned media (obtained from M1 culture and rich in pro-inflammatory cytokines), M2-conditioned media or a combination of both and quantify the cytokine and gene expression over time. This could give quantitative insight into the relative strength of the effect of M1 and M2 signaling and whether one polarization state dominates when both conditioned media are present.

As with all modeling approaches, the FBR model has certain limitations. Firstly, in our work we used a standardized ODE framework. This modeling choice was made to avoid any dependency on parameter values extracted from different experimental studies and the risk of biased results, due to the scarce amount of kinetic data. However, this required simplifying the system’s dynamics, which limited the model to (normalized) steady-state predictions and prevented accurate representation of temporal behavior. As shown by the sensitivity analysis, the standardized framework is not affected by changes in the network-related parameters, that were all set to the same baseline values. Although this is the purpose of the mathematical model used, we recognize that it is not entirely realistic, as we expect some factors to have a greater impact than others. While we demonstrated that the effects of B and C remain robust across different parameter variations, all parameters in the network would still benefit from further data to be more precisely defined and implemented in a classic ODE model.

Secondly, the input variables lump together many aspects of the complex FBR process and peri-implant area. Specifically, A aggregates the early inflammatory stages, during which a blood clot (provisional matrix) is deposited at the tissue/material interface and tissue damage signals stimulate the release of cytokines. These cytokines attract immune cells, such as neutrophils and monocytes to the injury site, where the latter differentiate into macrophages [59]. The variable A is decreased by regenerative macrophages activity (M2) and by ECM deposition, whereas it is increased by pro-inflammatory macrophages (M1) in a feedback loop that can lead to sustained inflammation over time. B lumps the immunogenic properties of a generic implant material, influenced by material features such as wettability and topography, that affect the absorption of proteins and immune cells adhesion. The C variable lumps different intrinsic mechanoregulatory aspects, such as cell-driven contraction, and extrinsic mechanical signals, such as the stiffness of the implanted material, the architecture of fibrous scaffolds—including fiber and pore size, and the stiffness due to increased collagen deposition [60], [61].

Thirdly, all interactions were simplified to one of two possible actions: activation (sharp arrow) and inhibition (blunt arrow), neglecting how these actions could switch over time. For example, recent literature has shown either activation or inhibition effects of TGF-*β* on fibroblasts depending on different stages of wound healing [62]. Finally, we modeled the transition of fibroblasts to myofibroblasts as an irreversible activation process, implementing it as two alternative states. This means that in our model, high myofibroblast activation corresponds to a low fibroblast level. Consequently, for highly critical inputs (e.g. A>0.5 or C>0.5, results not shown), the low fibroblast activation should be interpreted as a reduced fibroblast-to-myofibroblast ratio rather than low fibroblast recruitment.

Today, advanced computational models (e.g., physiology-based pharmacokinetic models, adverse outcome pathways) are already used to predict safety outcomes in drug testing [63]. Similarly, *in silico* models are being developed to fine-tune specific biomaterial properties — such as optimizing Ca²⁺ ion release from CaP-based scaffolds [64] —by providing precise parameter ranges for experimental validation in the prototyping phase. Moreover, computational mechanics is used to predict how implanted materials influence the mechanical properties of surrounding tissues, as seen in studies on stress distribution around breast implants and disturbed flow near vascular implants. *In silico* testing can strengthen pre-clinical assessment by enhancing the translation of *in vitro* findings, optimizing experimental design, and reducing reliance on costly and time-consuming animal studies. By incorporating these predictions into the ISO 10993 [65] framework, researchers can refine material formulations, optimize device designs, and reduce the need for *in vivo* testing while ensuring compliance with regulatory safety standards.

To advance the practical application of the FBR model for the development and evaluation of novel implant materials, some challenges need to be addressed as outlined in the roadmap below. A first important challenge to address in the future is to map in vitro data to in silico data and in silico data to in vivo data. Specifically, experimental conditions need to be quantified and mapped to the model variables, e.g. the material properties to the input variable B. Hereto, we envision an experimental quantification of the relation between the input variables A, B, C and their effect on the upstream network variables. With this, the experimental data could be normalized and mapped to a 0–1 scale as input for the in silico FBR model. For example, unbiased high-throughput screening of material libraries, similar to the work of Rostam et al. on the effect of polymer chemistries on macrophage attachment and polarization would allow measuring a range of data across materials that could be normalized to a 0–1 scale for variable B [66]. A significant (sub)challenge herein will be to decouple the different aspects that are lumped in the input variables B and C (see limitation discussed above). Advanced microphysiological systems, focused on decoupling cell-material interactions, offer interesting avenues to disentangle and quantify these individual mechanical parameters [67]. Given detailed experimental quantification of the various aspects that are currently lumped in the input variables B and C, including their potential interactions, the FBR input variables could be extended to further improve the biological realism of the model and explore the integrated FBR response. Similarly, the FBR in silico model predictions need to be mapped to in vivo outcomes, e.g. the low/high ECM levels to experimentally measured capsule thicknesses, or the macrophage levels to (scaled) macrophages surface markers quantification and M1/M2 ratio measured in vivo. This first mapping would enable qualitative predictions and comparisons with specific implant materials and their fibrotic response.

A second step involves developing a non-normalized, quantitative version of the FBR model. Such ODE model would allow quantitative predictions of the variable values and consequently a quantification of the error, using e.g. RMSE or AIC [68]. Such error quantification will help prioritize potential future model extensions. We envision that a relevant extension would be to make a partial differential equation (PDE) model, thereby incorporating the spatial aspects of the FBR, including, among other things, the dimension of the implanted material, the effect of cytokine diffusion and cell migration within the peri-implant area and the spatial orientation of collagen fibers (similar to [69]). Such spatial model could also be coupled to a mechanoregulatory model, like [70], establishing an iterative feedback loop between the biological activity and the biomechanical response (predicted as local stresses and strains in the tissue). Hereto dedicated data are required such as an extensive mechanical characterization of the tissue and implant material. We envision that a coupled mechanoregulatory model could be used to simulate implantable medical devices more accurately, paving the way for untangling the experimentally observed effects of key design parameters — such as shape and size examined in [52] — and optimizing future implant designs. For example, it could be used to investigate the effect of different mesh configurations (varying mesh burden) on the FBR found in vivo [71]. In summary, future quantitative models, once calibrated and validated, could help decipher the causal link between *in vitro* responses and *in vivo* encapsulation outcomes, addressing the persistent challenge of establishing a robust correlation between the two [72] and aid in improved response classification and (personalized) risk assessment.

To conclude, this work shows that material-related inputs can and should be included in models specific to the FBR and that standardized models can be informative tools when the specific data are too limited to reliably estimate dynamic parameters. It provides new avenues for better understanding the FBR using *in silico* modelling, to aid research on bioactive biomaterials and better integrated implants.

## CRediT authorship contribution statement

**Matilde Marradi:** Writing – review & editing, Writing – original draft, Visualization, Methodology, Formal analysis, Data curation, Conceptualization. **Martijn van Griensven:** Writing – review & editing, Writing – original draft, Supervision. **Nick R.M. Beijer:** Writing – review & editing, Writing – original draft. **Jan de Boer:** Writing – review & editing, Writing – original draft. **Aurélie Carlier:** Writing – review & editing, Validation, Supervision, Resources, Project administration, Methodology, Funding acquisition, Formal analysis, Conceptualization.

## Declaration of Competing Interest

The authors declare that they have no known competing financial interests or personal relationships that could have appeared to influence the work reported in this paper.
