# Supplementary material for: Towards predicting implant-induced fibrosis: A standardized network model of macrophage-fibroblast interactions
Source: Comput Struct Biotechnol J. 2025 Jul 13;27:3251–63. doi: 10.1016/j.csbj.2025.07.022 (PMC12312070; doi:10.1016/j.csbj.2025.07.022)
Supplement: Supplementary file 1 — Supplementary material [file mmc1.docx]

**Supplementary Material**

Table S1. **References.** References used for the construction of the biological network. Where not mentioned otherwise, studies specifically on the topic of FBR where used.

| **Interaction** | **References** | **Type of paper** | **FBR specific review** |
| --- | --- | --- | --- |
| Cytokines that induce M1 and M2 polarization | [1],[2],[3] | Review,  In vitro,  In vitro | Yes |
| M2 produced cytokines and proteins | [1] | Review | Yes |
| M1 produced cytokines and proteins | [4] | Review | Yes |
| IL10 effect on TGF$\beta$ | [5] , [6], [7] | Review,  In vitro, in vivo | Fibrosis  Pulmonary fibrosis, kidney fibrosis |
| IL10 anti-inflammatory effect on M1 | [4], [7],[8] | Review,  In vivo, in vitro | Yes,  kidney fibrosis |
| TGF$\beta$ on IL6 | [9],[10],[11] | Review,  In vitro,  In vitro | Skin fibrosis |
| PDGF effect on myofibroblasts | [12],[13] | Review,  in vitro | Yes |
| TGF$\beta$ effect on myofibroblasts | [14],[15],[16] | Review,  In vitro,  In vitro | Yes |
| Myofibroblast secretion of TGF$\beta$ | [17],[18],[19] | Review,  In vitro,  In vitro | Yes |
| Myofibroblast increased secretion of ECM components | [20],[19] | Review,  In vitro | Yes |
| Effect of stiff matrix on myofibroblasts | [21],[22],[23] | Review, In vitro,  In vitro | Fibrosis |
| Effect of stiff matrix on TGF$\beta$ | [24],[25],[26] | Review, In vitro,  In vitro | Fibrosis |
| Effect of IL1b on myofibroblasts | [27, 28] | Review, In vivo | Fibrosis,  Systemic sclerosis |
| Fibroblast secretion of MMPs | [29], [30] | Review, In vitro | Fibrosis |
| TIMPs and generic MMPs activity on ECM | [31],[32],[30] | Review, In vitro,  In vitro | Fibrosis |

*Figure S1* – Steady state values of all variables


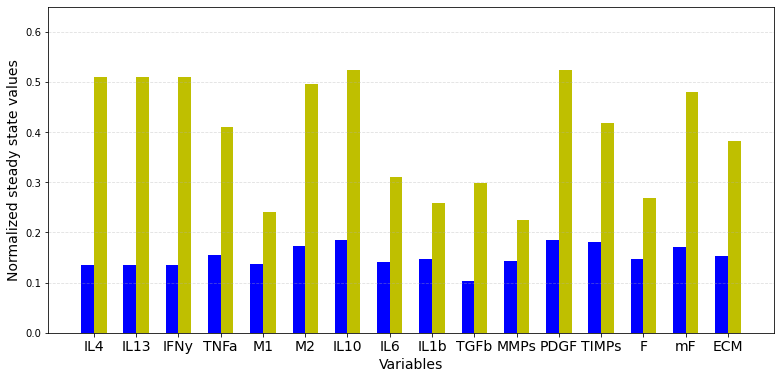


Figure S1. **Effect of increasing the starting value of input A – results for all variables in the network.** Blue: A(0) =0.3, Yellow A(0)=0.9. In both cases B=C=0. The normalized value at steady state between 0 and 1 represents the normalized concentration level (see Methods section 2.2).

Table S2. **Criteria for experimental study selection for perturbation simulations**. See Results Section 3.2.

| **Criteria for inclusion** | **Reason** |
| --- | --- |
| *In vivo* experiment | To evaluate if the integrated, tissue level outcome is comparable with the FBR model prediction |
| Soft tissue application | To exclude studies focusing on bone and cartilage, where FBR involves different cell types and fibrotic mechanisms |
| Experimental data for at least 14 days | To compare with the FBR model predicted steady state |
| No material degradation in the observed period | To enable comparison with the FBR model where this phenomenon is not included |
| ”binary” perturbations | To compare with the knock-out tests that can be performed with the FBR model |
| Perturbation applied to (at least) one of the upstream variables included in the FBR model | To enable to simulate a similar perturbation in the FBR model |
| Measurements/qualitative indication of variation in capsule thickness or ECM density | To compare with the predicted ECM levels |

Table S3. **Summary of the in vivo experiments used for the perturbation studies**.

For each in vivo experiment replicated, the table shows information extracted from the Methods section of the corresponding publications. NM: not explicitly mentioned in the Methods section

| **In vivo test** | **Species, detail** | **Number of replicates** | **Sex, age** | **Tissue** | **Time points** | **Way of measuring collagen content** |
| --- | --- | --- | --- | --- | --- | --- |
| IL-10 supplementation [33] | Wildtype mice, treated with PBS control or bleomycin concentration of 1500–2000 international units/kg weight intratracheally | NM | NM, 2-to-4 week old | Lung | Day 21 | Sircol™ Soluble Collagen Assay |
| PDGF receptor blocker [34] | Wild-type C57BL/6J and mice B6. 129S4-*Pdgfra^tm11(EGFP)Sor^*/J (PDGFRα-GFP) | NM | NM | Skin | Day 14 | Picro-Sirius red-stained histological sections  (5 µm thick skin samples) - hydroxyproline content in 8 mm skin punch biopsies |
| MMP-8 inhibition [35] | Wildtype and Mmp82/2 mice generated in  a mixed C57/Sv129 background | 106 | Male,  8-week-old | Lung | Days 3, 21, 42 | Sircol™ Soluble Collagen Assay |
| IL-4 eluting coating [36] | Wildtype C57BL/6J mice | 7 per group | Female, 8-10 weeks old | Subcutaneous implantation | Days 7, 14, 90 | Masson's trichrome and Picro Sirius Red staining |
| TGF-β inhibitor [37] | Wistar rats | 20 | Female, NM | Submammary implantation | Day 56 | Hematoxylin and Eosin (H&E) and Masson's Trichome staining (5-μm-thick sections) |
| IFN-y gene disruption [38] | Wildtype C57BL/6 mice (and with genetic deletion of IFN-**γ** (IFN-γ^−/−^) ) | 14 | Male, 7-8 weeks old | Subcutaneous implantation | Day 7 | Picro-Sirius red-stained histological sections |
| Inhibitory drug coating [39] | Wistar rats | NM | Female, adult | Subcutaneous implantation | Days 7,14,21,45 | Hematoxylin and Eosin (H&E) and Masson's Trichome staining |
| M depletion [40] | Wildtype C57BL/6 mice | 4 per group | NM, 8 weeks | Subcutaneous implantation | Days 7,21,35,60 | Second harmonic generation quantification |

*Table S4*. **Summary of the perturbation studies**.
The table shows the in vivo experimental conditions that were replicated with the model and the results obtained in terms of decreased or increased ECM value at the steady state. Arrow down: the perturbation induced decreased ECM activation level with respect to baseline in the in silico model. Arrow up: the perturbation induced increased ECM activation level with respect to baseline in the in silico model. Tick: in silico results matched in vivo findings. Cross: in silico results did not match. NC : in silico results could not be compared to in vivo findings, it was used for cases in which the in vivo experiments were performed on fibrotic diseases not induced by materials, therefore only input A was used. See paragraph 3.1 for detail.

| **In vivo test** | | **Material** | **Model perturbation** | **Input condition** | | | | | |
| --- | --- | --- | --- | --- | --- | --- | --- | --- | --- |
|  |  |  |  | A=0.5, B=0,  C=0 | A=0.5,  B=0.5,  C=0 | A=0.5, B=0, C=0.5 | A=0.2, B=0, C=1 | A=0.2, B=0, C=1 | A=0.2, B=0, C=1 |
| 1 | IL-10 supplementation [33] | No | IL-10=1 | **↓**  ✔ | **↓**  NC | **↓**  NC | **↓**  ✔ | **↓**  NC | **↓**  NC |
| 2 | PDGF receptor blocker [34] | No | PDGF=0 | **↓**  ✔ | **↓**  NC | **↓**  NC | **↓**  ✔ | **↓**  NC | **↓**  NC |
| 3 | MMP-8 inhibition [35] | No | MMPs=0 | **↑**  x | **↑**  NC | **↑**  NC | **↑**  x | **↑**  NC | **↑**  NC |
| 4 | IL-4 eluting coating [36] | Yes | IL-4=1 | **↓**  x | **↓**  x | **↓**  x | **↓**  x | **↓**  x | **↓**  x |
| 5 | TGF-β inhibitor [37] | Yes | TGF-β=0 | **↓**  ✔ | **↓**  ✔ | **↓**  ✔ | **↓**  ✔ | **↓**  ✔ | **↓**  ✔ |
| 6 | IFN-y gene disruption [38] | Yes | IFN-γ=0 | **↓**  ✔ | **↓**  ✔ | **↓**  ✔ | **↓**  ✔ | **↓**  ✔ | **↓**  ✔ |
| 7 | Inhibitory drug coating [39] | Yes | IL1,IFNy, TNFa, IL6=0 | **↓**  ✔ | **↓**  ✔ | **↓**  ✔ | **↓**  ✔ | **↓**  ✔ | **↓**  ✔ |
| 8 | M depletion [40] | Yes | M1,M2=0 | **↓**  ✔ | **↓**  ✔ | **↓**  ✔ | **↓**  ✔ | **↓**  ✔ | **↓**  ✔ |

*Figure S2* – ECM values for macrophage depletion


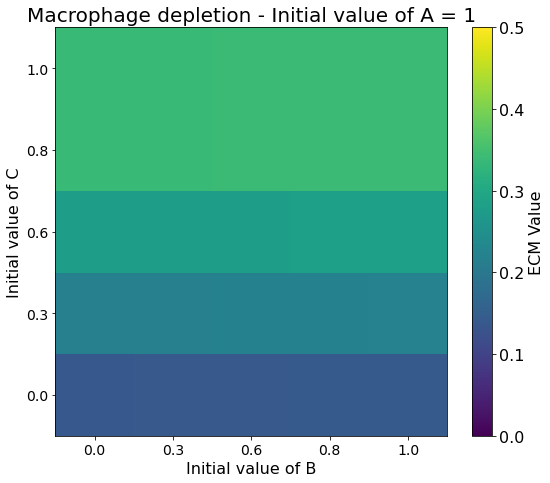


Figure S2. **Steady state values of ECM for A=1 and varying values of B and C in the case of macrophage depletion**. Macrophage variables M1 and M2 were set constant equal 0 throughout the entire simulation.

*Figure S3* – Heatmaps of ECM values


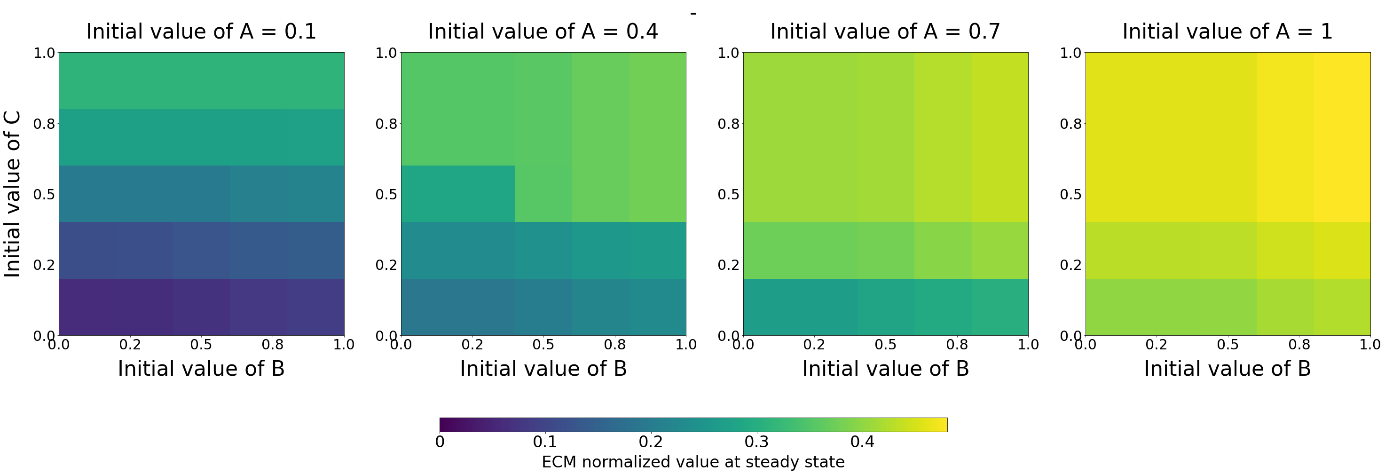


Figure S3. **Values of ECM for different initial values of input variables.** Each map corresponds to a different initial value of input A, from the left : 0.1, 0.4, 0.7, 1. In all maps, material-related input B increases along x and C along y. Pathological threshold of ECM activation levels is set to 0.36.

*Figure S4* - Heatmaps of ECM values for the model without feedback loops


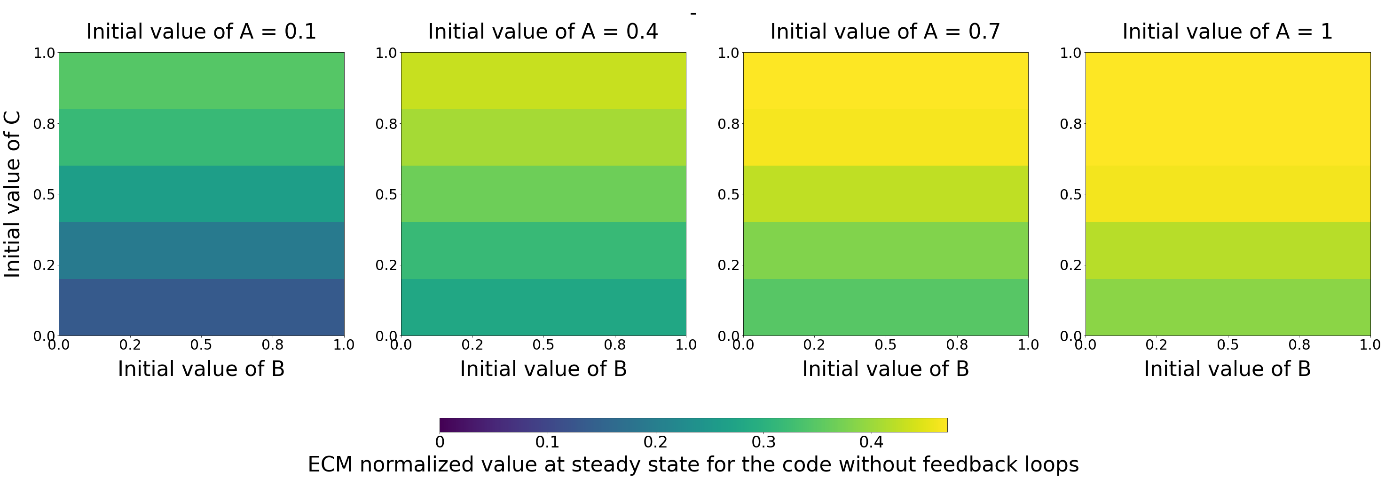


Figure S4. **Values of ECM for different initial values of input variables obtained from the code without feedback loops on input variables.** Each map corresponds to a different initial value of input A, from the left : 0.1, 0.5, 1. In all maps, material-related input B increases along x and C along y. Pathological threshold of ECM activation levels is set to 0.43.

*Figure S5*–Network-related parameters


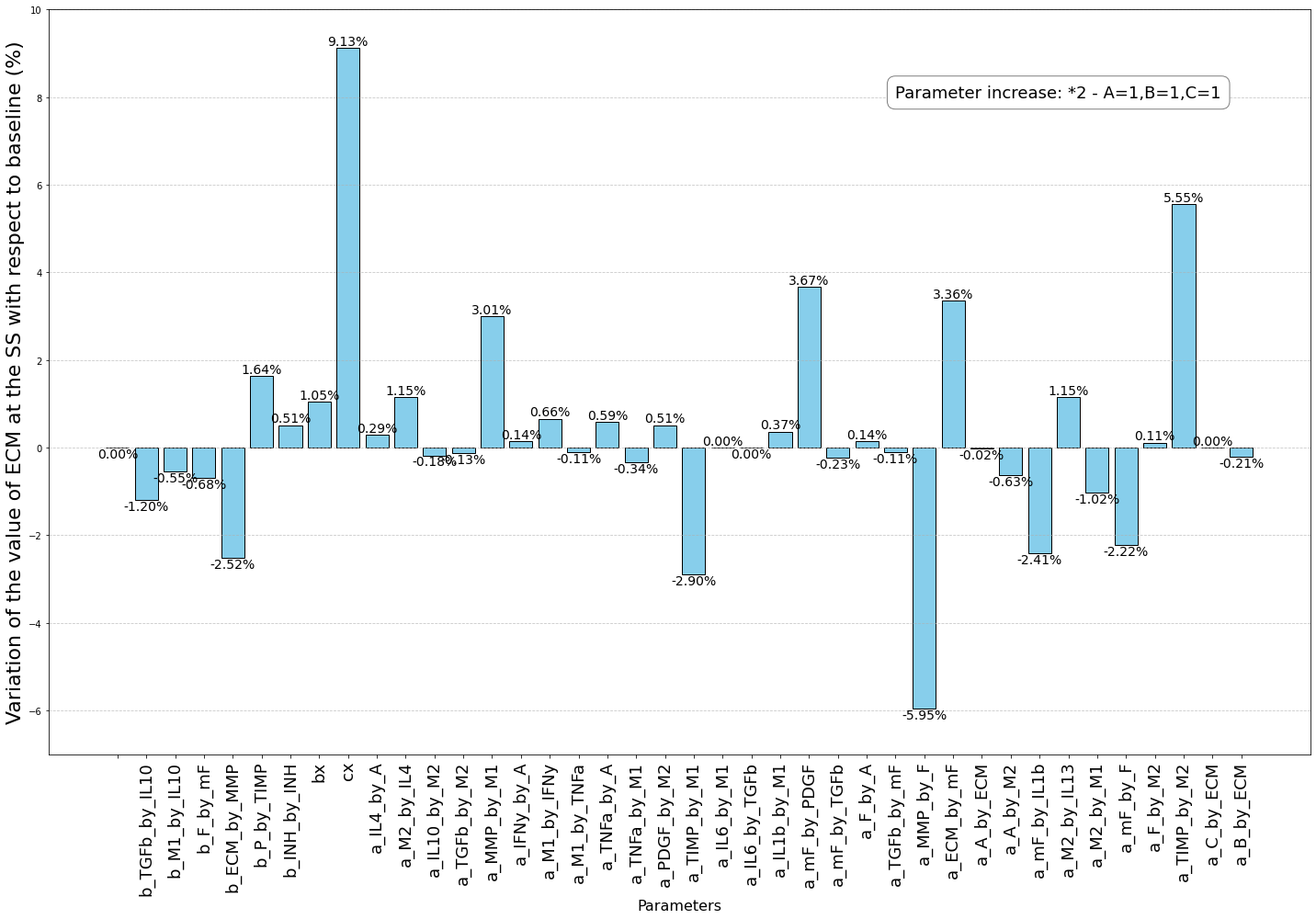


Figure S5. **Sensitivity of ECM value at the steady state to twofold variation of parameter values within the standardized framework**. Parameter names: a_affectedvariable_by_activatoryvariable, b_affectedvariable_by_inhibitoryvariable, bx:relative weight parameter value of the variable B, cx:relative weight parameter value of the variable C. For more detail on the names of all parameter values, we refer to the Github repository.

*Figure S6* – Input-related parameters


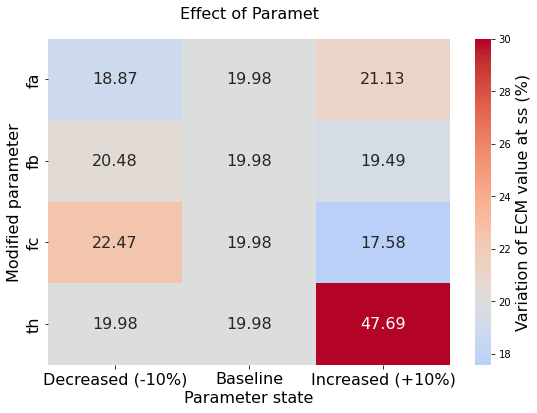


Figure S6. **Sensitivity of the effect of input variables B and C to parameter values changes.** The quantity of interest variation of ECM values is obtained by computing the difference between condition A=1, B=1, C=1 and A=1, B=0, C=0. Parameters are varied of +-10%

*Figure S7* – Heatmaps of ECM values at the steady state for different values of h


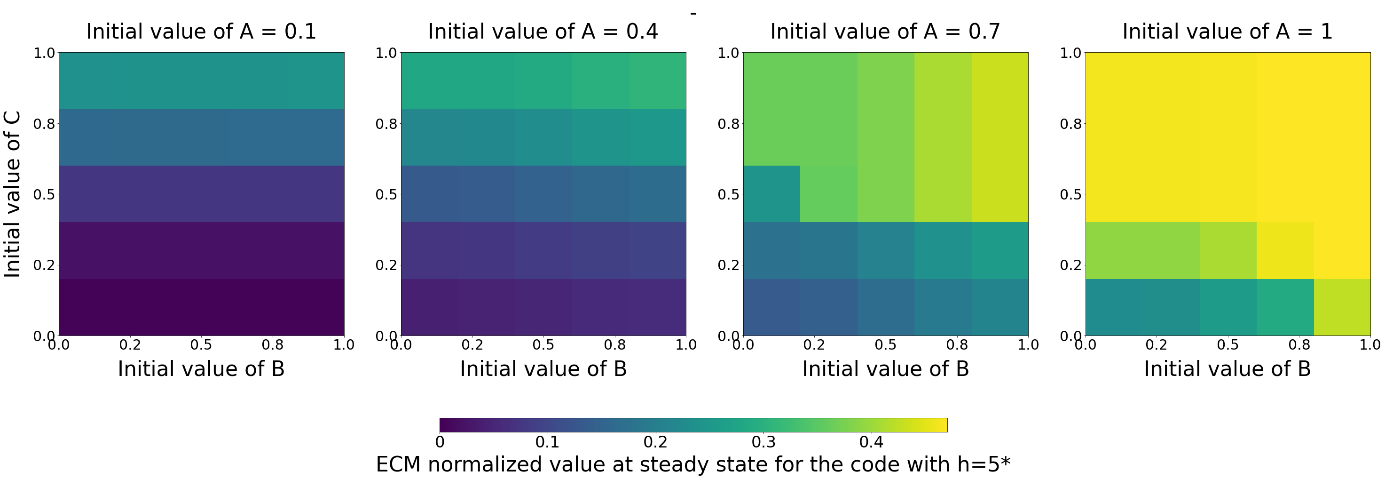


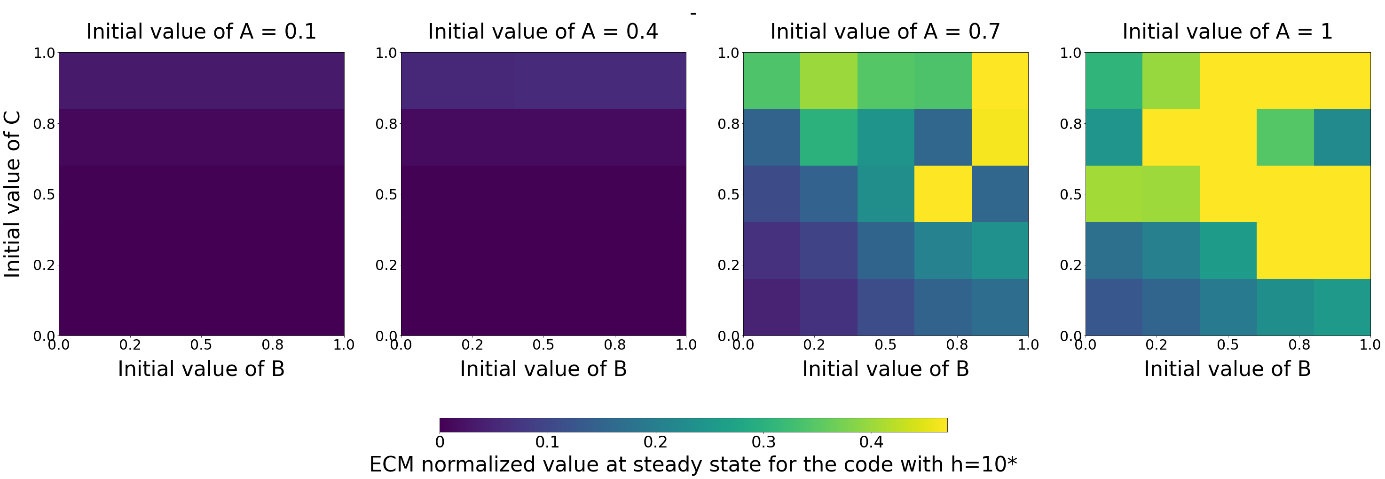


Figure S7. **Heatmaps of ECM values at the steady state for increasing values of h.** The gain parameter from Equation 1 of the mathematical framework was increased from the baseline value of 1 to 5, 10. ECM values are more polarized towards either 0 or 1.

1. Klopfleisch, R. and F. Jung, *The pathology of the foreign body reaction against biomaterials.* Journal of Biomedical Materials Research Part A, 2017. **105**(3): p. 927-940.

2. DeFife, K.M., et al., *Interleukin-13 induces human monocyte/macrophage fusion and macrophage mannose receptor expression.* The Journal of Immunology, 1997. **158**(7): p. 3385-3390.

3. Nathan, C.F., H.W. Murray, M.E. Wiebe, and B.Y. Rubin, *Identification of interferon-gamma as the lymphokine that activates human macrophage oxidative metabolism and antimicrobial activity.* Journal of Experimental Medicine, 1983. **158**(3): p. 670-689.

4. Kim, A., et al., *Investigating Immunomodulatory Biomaterials for Preventing the Foreign Body Response.* Bioengineering, 2023. **10**(12): p. 1411.

5. Sziksz, E., et al., *Fibrosis Related Inflammatory Mediators: Role of the IL-10 Cytokine Family.* Mediators Inflamm, 2015. **2015**: p. 764641.

6. Nakagome, K., *In vivo IL-10 gene delivery attenuates bleomycin induced pulmonary fibrosis by inhibiting the production and activation of TGF-  in the lung.* Thorax, 2006. **61**(10): p. 886-894.

7. Jin, Y., et al., *Interleukin-10 deficiency aggravates kidney inflammation and fibrosis in the unilateral ureteral obstruction mouse model.* Laboratory Investigation, 2013. **93**(7): p. 801-811.

8. de Waal Malefyt, R., et al., *Interleukin 10(IL-10) inhibits cytokine synthesis by human monocytes: an autoregulatory role of IL-10 produced by monocytes.* Journal of Experimental Medicine, 1991. **174**(5): p. 1209-1220.

9. Johnson, B.Z., et al., *The Role of IL-6 in Skin Fibrosis and Cutaneous Wound Healing.* Biomedicines, 2020. **8**(5): p. 101.

10. Eickelberg, O., et al., *Transforming growth factor-beta1 induces interleukin-6 expression via activating protein-1 consisting of JunD homodimers in primary human lung fibroblasts.* J Biol Chem, 1999. **274**(18): p. 12933-8.

11. Mazzarelli, P., et al., *Effect of transforming growth factor-β1 on interleukin-6 secretion in human myoblasts.* Journal of Neuroimmunology, 1998. **87**(1): p. 185-188.

12. Carnicer-Lombarte, A., S.T. Chen, G.G. Malliaras, and D.G. Barone, *Foreign Body Reaction to Implanted Biomaterials and Its Impact in Nerve Neuroprosthetics.* Front Bioeng Biotechnol, 2021. **9**: p. 622524.

13. Bonner, J.C., A.R. Osornio-Vargas, A. Badgett, and A.R. Brody, *Differential Proliferation of Rat Lung Fibroblasts Induced by the Platelet-derived Growth Factor-AA, -AB, and -BB Isoforms Secreted by Rat Alveolar Macrophages.* American Journal of Respiratory Cell and Molecular Biology, 1991. **5**(6): p. 539-547.

14. Witherel, C.E., D. Abebayehu, T.H. Barker, and K.L. Spiller, *Macrophage and Fibroblast Interactions in Biomaterial-Mediated Fibrosis.* Adv Healthc Mater, 2019. **8**(4): p. e1801451.

15. Ignotz, R.A. and J. Massagué, *Transforming growth factor-beta stimulates the expression of fibronectin and collagen and their incorporation into the extracellular matrix.* Journal of Biological Chemistry, 1986. **261**(9): p. 4337-4345.

16. Desmoulière, A., A. Geinoz, F. Gabbiani, and G. Gabbiani, *Transforming growth factor-beta 1 induces alpha-smooth muscle actin expression in granulation tissue myofibroblasts and in quiescent and growing cultured fibroblasts.* Journal of Cell Biology, 1993. **122**(1): p. 103-111.

17. Noskovicova, N., B. Hinz, and P. Pakshir, *Implant Fibrosis and the Underappreciated Role of Myofibroblasts in the Foreign Body Reaction.* Cells, 2021. **10**(7): p. 1794.

18. Damanik, F.F.R., et al., *Towards an in vitro model mimicking the foreign body response: tailoring the surface properties of biomaterials to modulate extracellular matrix.* Scientific Reports, 2014. **4**(1): p. 6325.

19. Juhl, P., et al., *Dermal fibroblasts have different extracellular matrix profiles induced by TGF-β, PDGF and IL-6 in a model for skin fibrosis.* Scientific Reports, 2020. **10**(1): p. 17300.

20. Li, R., et al., *Macrophages and fibroblasts in foreign body reactions: How mechanical cues drive cell functions?* Materials Today Bio, 2023. **22**: p. 100783.

21. Pakshir, P. and B. Hinz, *The big five in fibrosis: Macrophages, myofibroblasts, matrix, mechanics, and miscommunication.* Matrix Biology, 2018. **68-69**: p. 81-93.

22. Sharma, S., et al., *TRPV4 ion channel is a novel regulator of dermal myofibroblast differentiation.* Am J Physiol Cell Physiol, 2017. **312**(5): p. C562-c572.

23. McLane, Joshua S. and Lee A. Ligon, *Palladin Mediates Stiffness-Induced Fibroblast Activation in the Tumor Microenvironment.* Biophysical Journal, 2015. **109**(2): p. 249-264.

24. Wells, R.G., *Tissue mechanics and fibrosis.* Biochimica et Biophysica Acta (BBA) - Molecular Basis of Disease, 2013. **1832**(7): p. 884-890.

25. Achterberg, V.F., et al., *The Nano-Scale Mechanical Properties of the Extracellular Matrix Regulate Dermal Fibroblast Function.* Journal of Investigative Dermatology, 2014. **134**(7): p. 1862-1872.

26. Peters, A.S., G. Brunner, T. Krieg, and B. Eckes, *Cyclic mechanical strain induces TGFβ1-signalling in dermal fibroblasts embedded in a 3D collagen lattice.* Archives of Dermatological Research, 2015. **307**(2): p. 191-197.

27. Borthwick, L.A., *The IL-1 cytokine family and its role in inflammation and fibrosis in the lung.* Semin Immunopathol, 2016. **38**(4): p. 517-34.

28. Park, J.H., et al., *Materials and extracellular matrix rigidity highlighted in tissue damages and diseases: Implication for biomaterials design and therapeutic targets.* Bioact Mater, 2023. **20**: p. 381-403.

29. Cabral-Pacheco, G.A., et al., *The Roles of Matrix Metalloproteinases and Their Inhibitors in Human Diseases.* Int J Mol Sci, 2020. **21**(24).

30. Kobayashi, T., S. Hattori, and H. Shinkai, *Matrix metalloproteinases-2 and -9 are secreted from human fibroblasts.* Acta Derm Venereol, 2003. **83**(2): p. 105-7.

31. Brew, K. and H. Nagase, *The tissue inhibitors of metalloproteinases (TIMPs): an ancient family with structural and functional diversity.* Biochim Biophys Acta, 2010. **1803**(1): p. 55-71.

32. Sand, J.M., et al., *MMP Mediated Degradation of Type IV Collagen Alpha 1 and Alpha 3 Chains Reflects Basement Membrane Remodeling in Experimental and Clinical Fibrosis – Validation of Two Novel Biomarker Assays.* PLOS ONE, 2013. **8**(12): p. e84934.

33. Shamskhou, E.A., et al., *Hydrogel-based delivery of Il-10 improves treatment of bleomycin-induced lung fibrosis in mice.* Biomaterials, 2019. **203**: p. 52-62.

34. Makino, K., et al., *Blockade of PDGF Receptors by Crenolanib Has Therapeutic Effect in Patient Fibroblasts and in Preclinical Models of Systemic Sclerosis.* Journal of Investigative Dermatology, 2017. **137**(8): p. 1671-1681.

35. García-Prieto, E., et al., *Resistance to Bleomycin-Induced Lung Fibrosis in MMP-8 Deficient Mice Is Mediated by Interleukin-10.* PLoS ONE, 2010. **5**(10): p. e13242.

36. Hachim, D., S.T. Lopresti, C.C. Yates, and B.N. Brown, *Shifts in macrophage phenotype at the biomaterial interface via IL-4 eluting coatings are associated with improved implant integration.* Biomaterials, 2017. **112**: p. 95-107.

37. Gancedo, M., et al., *Pirfenidone Prevents Capsular Contracture After Mammary Implantation.* Aesthetic Plastic Surgery, 2008. **32**(1): p. 32-40.

38. Cassini‐Vieira, P., et al., *Lack of interferon‐gamma attenuates foreign body reaction to subcutaneous implants in mice.* Journal of Biomedical Materials Research Part A, 2018. **106**(8): p. 2243-2250.

39. Pakshir, P., et al., *Controlled release of low-molecular weight, polymer-free corticosteroid coatings suppresses fibrotic encapsulation of implanted medical devices.* Biomaterials, 2022. **286**: p. 121586.

40. Parlani, M., et al., *Dissecting the recruitment and self-organization of αSMA-positive fibroblasts in the foreign body response.* Science Advances, 2022. **8**(51): p. eadd0014.
